# Idea Technology and Ideology

**DOI:** 10.1007/s11013-021-09712-x

**Published:** 2021-03-09

**Authors:** Barry Schwartz

**Affiliations:** University of California, Berkeley, Berkeley, USA

**Keywords:** Idea technology, Ideology, Self-fulfilling prophesy, Neuroscience

## Abstract

Although we are accustomed to thinking about technology as involving things—objects and processes—derived from scientific discoveries, science also creates a technology of ideas, ways of thinking both about the world and about human beings. And unlike “thing technology,” “idea technology” can have powerful effects even when the ideas are false. This paper discusses false idea technology, or ideology, and suggests mechanisms by which it can have effects on both individuals and societies. It discusses neuroscience as the “next frontier” of ideology that may change our conceptions of human nature.

We are, in sum, incomplete or unfinished animals who complete or finish ourselves through culture—and not through culture in general but through highly particular forms of it.

—Clifford Geertz (1973:50)

“His brain made him do it.”

—Hon. Jed S. Rakoff, Senior United States District Judge of the United States District Court for the Southern District of New York, in a lecture at Swarthmore College, 2011.

## Introduction

On the campus where I used to teach, every time a new building was built or an old one was substantially renovated, an issue arose about where to locate the asphalt walkways that would go between that building and other campus locations. One school of thought suggested that the placement of walkways should be part of the building plan. But a second school, no doubt having observed many asphalt paths that lay unused near trails of dirt where once there had been grass, had the view that you build the building, watch where people walk, and put the asphalt where the grass has been worn thin. Proponents of the first view are folks we might call “theory driven.” Guided by some sense of efficient movement, esthetics, or both, they are inclined to do the “ideal” thing and have people conform to it. Proponents of the second view are folks we might call “data driven.” They let the users of the space tell them, with their behavior, what the “ideal” thing is.

When done right, all of science is an ongoing conversation between theory and data. The point of theories in science is to organize and explain the facts. Facts without organizing theories are close to useless. But theories must ultimately be accountable to the facts. And new facts force us to modify or discard inadequate theories.

That is the ideal. But in real life, things do not always work out this way. At least in the social sciences, theories, rather than being beholden to facts, can shape facts in a way that strengthens the theories. You build that path and then force people to walk on it, perhaps by roping off the grass.

This can be true even in the pristine world of the laboratory. An investigator publishes a paper that reports a significant finding using novel research methods. The paper attracts a good deal of attention. Others develop and extend the initial findings using the same methods. Quickly, a research tradition develops in which these particular methods are used to study this particular phenomenon. We now have a well-supported theory confirmed by a large set of empirical facts. But, alas, it turns out that the empirical methods on which this theoretical edifice is built are deeply flawed. There are confounds in the experimental designs, or questionable methods used in data gathering and analysis. Redone more carefully, new experiments fail to replicate the findings that created this edifice in the first place (see Simmons, Nelson, and Simensohn 2011). Thus, science may eventually and ultimately correct its mistakes, but disciplinary norms for conducting and analyzing research can perpetuate those mistakes for quite some time, making questionable results and incorrect theories seem self-evident to participants in a field until the mistakes are uncovered. Scientists operating at their best know (better than non-scientists) that all theories are false, i.e., provisional. Their hope is that their current mistaken efforts will at least be useful, rather than complete blind alleys (see Sterman 2002).

“If you build it, they will come.” This is the mantra that the main character in the movie *Field of Dreams* keeps hearing as he turns his farmland into a baseball park in the middle of nowhere. He builds it, and they *do* come. In this paper, I will try to show that at least sometimes, when social scientists build theories, the people come. That is, the people are nudged into behaving in ways that support the theories. This paper, then, is an attempt to resolve a conversation between metaphors. The “watch where they walk, then pave it” metaphor argues that the empirical data shape the theories people develop. The “if you build it, they will come” metaphor argues that theories shape data. I will attempt to defend the second metaphor.

The debate here is one that has been going on in more familiar territory for years. Does the market cater to consumer desires or does it create consumer desires? Do the media cater to people’s tastes in news and entertainment or do the media create those tastes? We are all accustomed to the difficulties surrounding discussion of these issues in modern society, and we may all have fairly strong opinions about the cater/create debate. Questions of just this sort are all around us.

In a sense, the distinction I am making is between discovery and invention. Discoveries tell us things about how the world works. Inventions use those discoveries to create objects or processes that make the world work differently. The discovery of pathogens leads to the invention of antibiotics. The discovery of nuclear energy leads to bombs, power plants, and medical procedures. The discovery of the genome leads, or will lead, to untold changes in almost every part of our lives. Of course, discoveries *also* change the world, by changing how we understand it and live in it, but they rarely change the world by themselves.

The distinction between discovery and invention is crucial. When a scientist, or anyone else, discovers something, it does not occur to us to ask whether that discovery *should* exist. In other words, although discoveries often have moral implications, they do not, by themselves, have moral dimensions. If someone were to suggest that the Higgs boson should not exist, we would wonder what mind-altering substance he had ingested. Inventions, in contrast, are a whole other story. Inventions characteristically have moral dimensions. We routinely ask whether they should exist. We wonder what is good (life improving) about them, and what the drawbacks are. We debate whether their wide distribution should go forward, and if so, with what kind of regulation.

So, is a theory about human nature a discovery, or is it an invention? I believe that often, it is more invention than discovery. For example, consider this, from Adam Smith:

It is in the inherent interest of every man to live as much at his ease as he can; and if his emoluments are to be precisely the same whether he does or does not perform some very laborious duty, to perform it in as careless and slovenly a manner that authority will permit. (1937:221)

I think that ideas like this from Smith, about what motivates people to work, have shaped the nature of the workplace (see Schwartz 2015). As noted by distinguished economist John Maynard Keynes:

The ideas of economists and political philosophers, both when they are right and when they are wrong, are more powerful than is commonly understood. Indeed the world is ruled by little else. Practical men, who believe themselves to be quite exempt from any intellectual influences, are usually the slaves of some defunct economist. (1965:386)

The ideas that Keynes is talking about are ideas about human nature—about what people care about, and what they aspire to. And like fish that do not know they live in water, we live with such ideas about human nature that are so pervasive that we do not even realize there is another way to look at ourselves. Where once our ideas about ourselves may have come from our parents, our community leaders, and our religious texts, these days, they come mostly from science—specifically from social science. Social science has created a “technology” of ideas about human nature.

Adam Smith’s ideas about human laziness helped give shape to the form the industrial revolution took. Ideas about racial differences in intelligence, self-discipline, and motivation helped give shape to the form that racial oppression took. Ideas about gender differences helped give shape to the form that gender discrimination took. And now, in the “age of the brain,” ideas about how neural processes determine our desires, thoughts, and actions promise to give rise to changes in how we think about human agency, autonomy, and responsibility (see Monterosso, Royzman, and Schwartz 2005; Monterosso and Schwartz 2020). Of all the technologies that humanity has produced over its history, the technology of ideas may be the most powerful.

## Idea Technology

We live in a culture and an age in which the influence of scientific technology is obvious and overwhelming. Whether it is laptops, smart phones and tablets, or MRI scans, gene modifications, and designer drugs, adjusting to technology is a brute and insistent fact of daily life. Some of us embrace technology enthusiastically, and some of us do it grudgingly, but everyone does it.

The technology of smart phones and MRIs—the technology of things—is what most of us think of when we think about the modern impact of science. But in addition to creating things, science creates concepts, ways of understanding the world, and our place in it, which have an enormous effect on how we think and act. If we understand birth defects as acts of God, we pray. If we understand them as acts of chance, we grit our teeth and roll the dice. If we understand them as the product of prenatal neglect, we take better care of pregnant women.

It hardly needs to be said that people are profoundly affected by the material conditions of their lives—by the affluence of the societies they inhabit. People without food will starve whether they accept their conditions beatifically as God’s will, accept them with depressed resignation as indications of their own inadequacy, or respond to them in anger at social injustice. No matter what ideas people appeal to explain their lack of food, their bellies remain empty.

And yet it is clear that ideas *also* matter, and they matter a lot, even in the case of an obvious material condition like the availability of food. What a squirrel foraging for food in the park does in times of scarcity has nothing to do with how the squirrel understands this scarcity. The squirrel is not about to pray for food, cultivate trees, or organize other squirrels to rise up in protest against people who have polluted the environment and diminished its food supply; the squirrel just forages for food. But what people do about their lack of food depends a great deal on how they understand it. Ideas have much to do with whether massive food shortages yield resignation or revolution.

If we understand the concept of “technology” broadly, as the use of human intelligence to create objects or processes that change the conditions of daily life, then it seems clear that ideas are no less products of technology than are computers. However, there are two things about idea technology that make it different from most “thing technology.” First, because ideas are not objects, to be seen, purchased, and touched, they can suffuse through the culture and have profound effects on people before they are even noticed. Second, ideas, unlike things, can have profound effects on people *even if the ideas are false*. Smart phones, designer drugs, and the like generally do not affect people’s lives unless they do what they were designed to do. Companies cannot sell technological objects that fail—at least not for very long. Technological objects may do bad things that people do not want them to do, but at least, there is little reason to worry about them unless they can also do the things they were designed to do in the first place. In contrast, false ideas can affect how people act, just as long as people believe them. Following philosophers Marx and Engels (Engels 1949), let us call instances of idea technology based on untrue ideas “ideology” (see Hanson 2012 for a discussion of varied uses of the term “ideology,” and Schwartz 1997, 2012, 2015 for discussions of “idea technology.”) Because idea technology often goes unnoticed, and because it can have profound effects even when it is false—when it is ideology—it is in some respects more profound in its influence than the thing technology whose effects people are so accustomed to worrying about.

Why are not ideas just like things? The hallmark of science is that it operates in the world of testable hypotheses. If you have an idea, you test it, and if it fails the test, it also disappears, just like failed thing technology. So, it would seem, there is no need to worry about a technology of false ideas. False ideas will just die of “natural causes.” Right?

Alas, no. For example, I think ideology bears a large measure of the responsibility for the nature of our work. Here is Adam Smith:

The man whose life is spent in a few simple operations . . . has no occasion to exert his understanding, or to exercise his invention in finding out expedients for difficulties which never occur. He naturally loses, therefore, the habit of such exertion and generally becomes as stupid and ignorant as it is possible for a human creature to be. (1937:782)

The key things to notice about this statement are the words “loses” and “becomes.” Here is Smith, the father of the assumption that people are basically lazy and work only for pay, saying that work in a factory will cause people to “lose” something, and “become” something. So, what is it that they *had* before entering the factory that they “lost”? And what is it that they *were* before entering the factory that was different from what they “became”? Right here in this quote, we see evidence that Smith believed that what people were like as workers depended on the conditions of their work. And yet, over the years, this nuanced understanding of human nature as the product of the human environment got lost.

And this loss was crucial. If we view Smith’s account as a “discovery” about human nature, then the soul-deadening workplaces that many people face needs no justification except for economic efficiency. It is not changing people. It is not depriving people of anything. It is simply taking people as they are and using their labor with maximum efficiency. Thus, we need not ask whether workplaces *should* be organized in this way.

We now know that the industrial workplace *was* changing people. A classic article by Kohn and Schooler (1982) showed as much. They showed that work over which people exercise some discretion and control leads to cognitive flexibility and to an engaged orientation to self and society; in contrast, excessively monitored, oppressively supervised working conditions lead to distress. More recently, in a similar vein, DeVoe and Pfeffer (2007) have shown that the way in which people are compensated changes them. Professionals who bill by the hour, like lawyers and consultants, start putting a price on their time, even when they are not at work. An evening spent with friends watching a ball game has “costs” in legal fees and consulting fees forgone. So, a person who bills by the hour becomes a different person than she was before she started working in that way.

Also striking is a series of studies by Heath (1999) that show that even when people do not think of *themselves* as primarily motivated by material incentives, they think that pretty much *everyone else* is. Heath surveyed students taking the Law School Admissions Test (LSAT). They were asked to describe their own motives for pursuing a legal career, and then to speculate about the motives of their peers. Sixty-four percent said that they were pursuing a legal career because it was intellectually appealing or because they had always been interested in the law, but only 12% thought that was true of their peers. Instead, 62% speculated that their peers were pursuing a legal career because of financial rewards. So, we may tell ourselves that we are exceptional in caring about things besides money, which in turn makes it easier for us to organize the work of others entirely based on monetary incentives.

Along similar lines, Heath (1999) reports results from the General Social Survey (GSS). For more than twenty-five years, the GSS has asked a sample of adults to rank the importance of five different aspects of their jobs: pay, security, free time, chances for advancement, and “important work” that “gives a feeling of accomplishment.” Year after year, “important work” is, on average, ranked first by more than 50% of the individual respondents. Pay typically ranks third. Yet, in the late 1980s, when the GSS asked respondents about the role of material incentives for others, people generally believed that pay was quite important.

So, ideas change people. The pressing question is: How can idea technology take root, even when the ideas are false—even when they are ideology?

## Ideology: Mechanisms

One might think that in the scientific, big-data age in which we live, eventually the empirical evidence will win the day, and good data will drive out false ideas. The history of science is a history of one mistaken theory after another, with careful collection and interpretation of data the engine for correcting these mistakes. Better data and theories drive out worse ones, and progress is made. Why does this not happen in the case of theories about human nature?

Well, sometimes it does. Psychologists have made much progress over the years in understanding perception, memory, thinking, language use and comprehension, cognitive and social development, learning, and various types of emotional and cognitive disorders in exactly the same way that natural sciences make progress in their domains. Good data drive out bad theories. But there is a crucial difference between theories about planets, atoms, genes, and diseases and theories about at least some aspects of human nature. Planets do not care what scientists say about their behavior. They move around the sun with complete indifference to how physicists and astronomers theorize about them. Genes are indifferent to our theories about them also. But this is not true of people. Theories about human nature can actually produce changes in how people behave (see Gergen 1973). What this means is that a theory that is false can *become* true simply by people believing it is true. As a result, instead of good data driving out bad data and theories, bad data can change social practices until the data become good data, and the theories are validated.

I think there are three basic dynamics through which false ideas can become true. The first way ideology becomes true is by *reconstrual*—by changing how people think about and understand their own actions. For example, someone who volunteered every week in a homeless shelter might one day read a book that tells him it is human nature to be selfish. He might then say to himself, “I thought I was acting altruistically. Now, social scientists are telling me that I work in a homeless shelter for ego gratification.” Or someone on her way to work might say, “I thought I showed up for work every day eager to be challenged and do a good job that improves someone’s life. Now social scientists are telling me it’s all about the money.” If this kind of reconstrual mechanism is acting, nothing outside the person necessarily changes. The person simply understands her actions differently. But of course, how we understand our past actions is likely to affect our future actions.

The second mechanism by which ideology becomes true is via what is called the *“self-fulfilling prophesy.”* Here, ideology changes how other people respond to the actor, which, in turn, changes what the actor does in the future. A classic demonstration of this self-fulfilling mechanism in action was reported by Snyder and Tanke (1977). In this study, groups of college men were shown a photo of either an attractive or an unattractive woman. They then had a ten-minute phone conversation about academic matters with a woman they were led to believe was the woman in the photo (she was not). After the conversation, those who thought they were talking to the attractive woman rated her as more likeable than those who thought they were talking to the unattractive woman. No surprise here. The surprise came next. Tapes of the conversations were played for other participants who had not seen photographs of the woman or been told anything about her attractiveness. They, too, judged the “attractive” woman as more likeable, friendly, and sociable than the unattractive one.

Think about this result. Somehow, thinking their interview subject was attractive led interviewers to conduct their interviews in a way that led third parties who listened to the interview to come to the same conclusion. In effect, the interviewers collected “data” in a way that was biased by their initial beliefs.

The phrase “self-fulfilling prophecy” was coined by sociologist Robert Merton in 1948. He discussed examples of how theories that initially do not describe the world accurately can become descriptive if they are acted upon. In essence, a “self-fulfilling prophesy” is, in the beginning, “a *false* definition of the situation evoking a new behavior that makes the originally false conception come *true*” (Merton 1948:196).

Merton went on to explore the causal link between a prophecy and its subsequent confirmation. For instance, Merton cited the then prevalent notion that black workers were “unsuitable” to be members of labor unions. With their “low standard of living” and willingness to “take jobs at less than prevailing wages,” these laborers became “traitors to the working class.” What the exclusionary unions failed to recognize, argued Merton, is that the very act of excluding black workers from the union caused the behavior that seems to confirm the hypothesis. Not being members of the union during a strike, black workers would cross the picket line to fill the labor void, breaking the strike, ostensibly vindicating the original claim as to their unsuitability for union membership. What was originally a false hypothesis—an ideology—changed the situation, configuring it in ways that appeared to validate it.

Another notable example of this process is the teacher who pays more attention and works harder with children identified as “smart” than children identified as “slow,” thereby making the “smart” ones smarter. Thus, being labeled as “smart” or “slow” does not in itself make kids smarter or slower. The teacher’s behavior must also change accordingly. Perhaps the best-known demonstration of the self-fulfilling prophesy in education is shown in the research conducted by Rosenthal and Jacobson (1968) on the effects of teacher expectations on student performance. Unbeknownst to the teachers in the study, the researchers randomly assigned certain students in an elementary school classroom to the “spurter” condition. These students supposedly had taken a diagnostic test at the end of the preceding school year that identified them as having the potential for impressive academic gains. No such test really suggested any difference between the spurters and their peers. Nonetheless, the students who had been labeled as spurters *did* manifest more impressive gains than average by the end of the school year. High expectations from the teacher somehow resulted in higher student achievement. In short, Rosenthal and Jacobson argued, the labeling of certain students as promising became a self-fulfilling prophecy by changing the way teachers taught. This finding has been highly influential in both the fields of psychology and education.

Years later, Lee Jussim and colleagues followed up on this line of inquiry by assessing specific ways in which teacher expectations affect student performance and the specific contexts in which such expectations have the most marked effects (Jussim 1986; Jussim 1989; Madon, Jussim, and Eccles 1997; Smith, Jussim, and Eccles 1999). Though they found evidence supporting the self-fulfilling prophecy framework laid out by Rosenthal and Jacobson, they also identified boundaries to the pervasiveness and power of the self-fulfilling prophecy. Jussim found that such prophecies are not all-pervading, and the magnitude of the effects, though significant, is often modest.

It is perhaps not surprising that these effects, when they occur, are not large. After all, the kids may get one subtle message about their ability in school but quite a different one at home from their doting parents. But if the message were delivered more consistently, across all the domains of a child’s experience, then the effects might be very large indeed.

Can such a pervasive message be sent? Well, think about the way in which many psychologists and educators talk about intelligence. There is some evidence, and much belief that individual differences in intelligence are innate and unmodifiable. Some people win the genetic lottery and some lose it. It is not hard to imagine that if this idea about intelligence became commonplace, parents would take their cue from teachers and give their kids the same messages the kids were getting in school. But is this understanding of intelligence true, or is it ideology?

Consider the work of psychologist Carol Dweck, summarized in her book *Mindset* (2006; see also Dweck and Leggett 1988). Dweck has discovered that we can distinguish among children based on the goals that seem to be operating while they learn. Some kids have what Dweck calls “performance” goals. They want to do well on tests. They want social approval. Other kids have what Dweck calls “mastery” goals. These kids want to encounter things that they cannot do and to learn from their failures. As Dweck puts it, performance-oriented children want to *prove* their ability while mastery-oriented children want to *improve* their ability.

Children with performance goals avoid challenges; children with mastery goals seek them. Children with performance goals respond to failure by giving up; children with mastery goals respond to failure by working harder. What this means is that children with mastery goals learn more, and get smarter, than children with performance goals.

Dweck has shown that what lies beneath these two orientations is a pair of quite different conceptions or “theories” children have of the nature of intelligence. Some children believe that intelligence is essentially immutable—that it is a fixed entity. These are the children who tend to be performance oriented. What is the point of seeking challenges and risking failure if you cannot get any smarter? Other children believe that intelligence is not fixed—that it is incremental, and people *can* get smarter. These children tend to be mastery oriented, seeking in their schoolwork to do what they believe is possible for everyone. So, is intelligence fixed? Partly, that depends on whether you believe it is fixed. What this means is that the theory that intelligence is fixed may well be ideology.

Not surprisingly, results parallel to Dweck’s have been found in the workplace. Heslin, Latham, and VandeWalle (2005) did a series of studies of how managers manage their employees. They discovered that managers also seemed to have either fixed or incremental theories of employee ability. If managers had a fixed theory of ability, they were less likely to notice changes in employee performance, and less likely to provide feedback and coaching aimed at improvement, than if they had an incremental theory of ability. What is the point, they seemed to think, of trying to improve something that cannot be changed. It is like trying to improve someone’s height by encouraging him to grow. But you can see the feedback loop that such an attitude creates. The manager does not think performance can be improved, so she does nothing to try to improve performance. Lo and behold, performance does not improve, and her theory is thereby confirmed.

It should be noted that as in the case of research on the self-fulfilling prophesy, recent research has called into question not the existence of “mindsets” or the claim that they may affect behavior, but the magnitude of the effects. For example, Sisk et al. (2018) did an analysis of extensive research on the effects of mindset on academic achievement and the effects of efforts to alter mindset on changes in academic achievement and found rather small effects—smaller than the effects of other academic variables and interventions. Some results did support claims of Dweck’s that students of low socioeconomic status or academically at risk might especially benefit from mindset interventions, but Sisk et al. argued that one should expect the effects to be quite modest.

To a large degree, the effects of ideology on how people act will depend on how broadly, how pervasively and how saliently it is purveyed in a culture. When it lives in isolated places, its effects will likely be small and correctable. But when it is in the water supply—when it is everywhere—its effects will likely be much more profound. In support of this point, psychologist Richard Nisbett (2009) has shown that entire cultures can be differentiated from one another by the extent to which they are guided by the belief that either intelligence is fixed or that it grows. And studies of intellectual development across these different cultures show that kids living in a culture with a “growth mindset” exhibit more intellectual development than kids living in a culture with a “fixed mindset.”

This brings us to the final mechanism by which ideology can have an influence. This mechanism—the one that I believe has the most profound effects on human beings—is when *institutional structures* are changed in a way that is consistent with the ideology. The industrialist believes that workers are only motivated to work by wages and then constructs an assembly line that reduces work to such meaningless bits that there is no reason to work aside from the wages. The politician believes that self-interest motivates all behavior, that people are entitled to keep the spoils of their labors, and that people deserve what they get and get what they deserve. Said politician helps enact policies that erode or destroy the social safety net. As a result, people start acting exclusively as self-interested individuals. “If it’s up to me to put a roof over our heads, put food on the table, and make sure there’s money to pay the doctor and the kids’ college tuition bills, then I’d better make sure I take care of myself.” When social structures are shaped by ideology, ideology can change the world.

An example of this type of pervasive ideology in action was offered by Ferraro, Pfeffer, and Sutton (2005) in a discussion of how theories of rational choice in economics come to shape the behavior of both individuals and institutions. In describing the development of trading practices on the Chicago Board Options Exchange (CBOE), they observe the following (Ferraro, Pfeffer, and Sutton 2005:12–13):

In a fascinating historical case study, MacKenzie and Millo (2003) studied the development of the CBOE, which opened in 1973 and quickly became one of the most important financial derivatives exchanges in the world. The same year the CBOE opened, Black and Scholes (1973) and Merton (1973) published what were to become the most influential treatments of option pricing theory, for which the authors were to win the Nobel Prize in Economics. The formula developed in this work expressed the price of an option as a function of observable parameters and of the unobservable volatility of the price of the underlying asset. It is important to note that this formula originally did not accurately predict option prices in the CBOE, with deviations of 30 to 40% common in the first months of option trading. Yet, as time passed, deviations from the model diminished substantially so that, for the period August 1976 to August 1978, deviations from the Black-Scholes price were only around 2% (Rubinstein 1985). This success in the theory’s predictions of option prices led Ross (1987) to characterize option pricing theory as “the most successful theory not only in finance, but in all of economics” (1987:332). MacKenzie and Millo (2003) showed that this increasing accuracy resulted because people and organizations acted as if the theory were true, which made its predictions come true. Interviews with market participants revealed, for example, that traders started to use the theoretical value sheets obtained from the Black-Scholes equation to determine their bids. The model also became increasingly institutionalized in the regulatory framework of the market, in its language, and in its technological infrastructure, especially in the Autoquote system software launched by the exchange in 1986 that implemented the Black-Scholes formula and provided traders with theoretical prices for all the options being traded. “Financial economics, then, helped create in reality the kind of markets it posited in theory” (MacKenzie and Millo 2003:54).

When ideology is embedded in social structures, we must be especially vigilant about identifying it. It is much harder to change social structures than it is to change how people think about themselves, which psychotherapy may effectively address, or how they think about others, which education may effectively address. Moreover, because social structures affect multitudes rather than individuals, when these structures embody ideology, the effects of that ideology can be pervasive.

Psychologist Dale Miller (1999) presented evidence of the pervasiveness of an ideology he calls the “norm of self-interest” in American society. College students assume, incorrectly, that women will have stronger views about abortion issues than men and that students under the age of twenty-one will have stronger views about the legal drinking age than those over twenty-one, because women and minors have a stake in those issues that men and older students do not. The possibility that one’s views could be shaped by conceptions of justice or fairness, rather than self-interest, does not occur to most people. And yet they are. Empathy and concern for the well-being of others are routine parts of most people’s character. Yet they are in danger of being crowded out by exclusive concern for self-interest—a concern enhanced by the ideology that self-interest is all there is.

A suggestion that ideology can operate on a society-wide scale comes from a content analysis of Norwegian newspapers (Nafstad et al. 2009). The analysis, which covered a period from 1984–2005, found a shift from what the authors call “traditional welfare ideology,” long the dominant sociopolitical characteristic of Norwegian society, to what they call “global capitalist ideology.” That shift included increased use of market-like analysis to discuss all aspects of life, increased reference to the values of individualism and self-interest, and a redefinition of the social contract between individuals and society along incentive-based lines. Of course, the fact that newspapers write about social relations in a particular way does not mean that people live them in that way, but it is at least plausible that newspaper coverage either captures a shift in how people think about and act toward one another, or facilitates such a shift, or both.

Even Adam Smith, the father of free-market capitalism, understood that there was more to human nature than self-interest. *The Wealth of Nations* followed another book, *The Theory of Moral Sentiments* (Smith 1976), in which he suggested that a certain natural sympathy for one’s fellow human beings provided needed restraints on what people would do if they were left free to “barter, truck, and exchange one thing for another.” Smith’s view, largely forgotten by modernity, was that efficient market transactions were parasitic on aspects of character developed through nonmarket social relations. Smith was right about the importance of “moral sentiments” but wrong about how “natural” they are. In a market-dominated society, in which every aspect of what people do is “incentivized,” these “moral sentiments” may disappear so that nothing can rein in self-interest.

## Can the Effects of Ideology Be Cyclical?

The three mechanisms I identified suggest that ideology can set a process in motion that will continue indefinitely unless impeded or redirected by another force. This may be so, but another possibility is that ideology’s effects may be somewhat self-correcting, and thus cyclical. Imagine, for example that a factory system develops that maximizes efficiency with minute division of labor, so that workers engage in the same simple, thoughtless activity for hour after hour, day after day, week after week. Before this process of routinization is “perfected,” people adjust to having less control and discretion, being less challenged, and getting less satisfaction out of their work. Such a process would be an illustration of ideology in action with the causal agent being the shape of the workplace, a major social institution. But as this process continues, a point may be reached at which workers refuse to be treated in this way, and demand a restructuring of the work they do. Such a reaction might serve to reverse the process of routinization, leading to some sort of equilibrium (perhaps temporary) in which work is not as fulfilling as it might be, but not as soul deadening either. A cyclicity of this sort has been suggested by Rand et al. (2017) in connection to the balance between automatized and reflective aspects of cognitive processing. There is no logical reason why cyclicity should not occur; whether it does is an empirical question (and see Gergen 1973, for a more general argument that whereas we can always expect ideology to have effects, the direction of those effects is not always predictable.)

## Ideology and Neuroscience

Recent developments in psychology have added a new and potentially quite powerful source of idea technology (I leave open whether it is also an example of ideology) to our understanding of human nature and ourselves. Psychology is coming increasingly to be dominated by the fields of neuroscience and genetics. Explanations of human behavior that appeal to psychological states and variables are coming to be seen as mere “holding actions,” awaiting “real” explanations that appeal to genes, brains, and nervous systems. And this conceptual change in psychology has filtered into more popular media. Colorful pictures of brain scans have become an almost routine part of reporting on psychology to the public. How, if at all, might this shift in psychological explanation affect the technology of ideas?

Consider this example. In 2005, the United States Court ruled the death penalty unconstitutional for juveniles. A significant rationale for this ruling was evidence from neuroscience that the brain’s frontal lobes (crucial for planning and self-control) are not yet mature in adolescents. This led to the belief that in an important sense, adolescents who do dangerous and violent things cannot help it; they cannot control themselves. Well, duh! It surely did not take neuroscience to affirm what specialists in adolescent development (and virtually all parents) have long known and what anyone who ever watches teenagers regards as self-evident—namely that teenagers as a group are immature risk takers. Yet, the neuroscience seems to have played a critical, maybe even decisive role in confirming what is obvious and in changing the Supreme Court’s, and society’s attitudes about the degree to which teenagers should be held responsible for their risk taking.

The issues at stake here extend far beyond the punishment of adolescents. If we take any sign of brain involvement in human behavior as a sign that the behavior was not under the individual’s control, an entire social system built on holding people responsible for the things they do will be challenged. So, it is important to think clearly about what neuroscience tells us about notions of responsibility before the science gets ahead of the social institutions on which we rely.

Human beings seem to understand the world in two fundamentally different ways. When we try to understand the material world, we do it by identifying *causes*. Causes explain behavior by reference to the *past*—to the antecedents from which the event followed. When we ask “what made the airplane lose altitude so fast as it was coming in for a landing?” we are seeking the cause of the airplane’s behavior. But when we try to understand one another, we rely on *intentions*, on *reasons*. Intentions explain behavior with reference to something desired in the *future*. When we ask “why is the pilot motioning to the co-pilot? What is he trying to get him to do?” we are asking about the pilot’s goals. The adoption of one of these two modes is so natural and automatic that the distinction is easy to miss. It would be silly to try to understand the behavior of airplanes, appliances, and storm clouds by considering their intentions (we do not really mean it when we say that our malfunctioning cell phone has been “out to get us.”) And we usually cannot help but take what the philosopher Daniel Dennett (e.g., 1978) calls the “intentional stance” when thinking about and interacting with other people.

For the most part, our division of the world into the material and the human goes smoothly. One of these two ways of explaining what we are interested in seems a good fit, and the other does not. But our changing attitude toward adolescent transgressions challenges this division. The immature brain of adolescents is a cause, not a reason. And research by Monterosso, Royzman, and Schwartz (2005) suggests that this is just the tip of a very big iceberg. For the lay reader, “my brain made me do it” has a plausibility that “psychological factors operating on my cognitive, emotional and motivational systems made me do it” does not.

To illustrate the power of explanations that appeal to neuroscience, Monterosso, Royzman, and Schwartz (2005) asked research participants to consider scenarios involving individuals who behaved in ways that caused harm to themselves or others, including acts of violence. We included information about the protagonist in the scenario that might help make sense of the action in question. In some cases, that information was about a history of sometimes horrific events that the individual had experienced (e.g., a history of severe abuse as a child), and in some cases, it was about characteristics or anomalies in the individual’s brain (e.g., an imbalance in neurotransmitters). We also varied information about the strength of the connection between these explanatory factors and the behavior, as well as how easily the behavior could be deterred.

The pattern of results was striking. A brain antecedent that was even weakly associated with violence (that is having effects of the same order of magnitude of most psychological variables) led people to exonerate the perpetrator more than an experiential/psychological antecedent (e.g., severe child abuse) that was strongly associated with violent acts. Moreover, respondents were much more likely, given a brain antecedent, to view the behavior as “automatic” rather than “motivated,” and to view the bad behavior as unrelated to character. In follow-up interviews, the participants in our study described the protagonists with the brain antecedent in ways that suggested that the “true” person—the intentional mind—was not at the helm. The behavior was *caused*, not *intended*. The person with a brain defect could no more help doing what he did, they seemed to be saying, than a dropped glass can help falling to the floor. “My neurotransmitters made me do it.” In contrast, whereas experiential antecedents like childhood abuse often elicited sympathy, and sometimes even considerable mitigation of blame, this path appeared qualitatively different. The experiential explanation did not lead our informants to abandon the intentional stance people typically take toward one another. The perpetrator *himself* was twisted by his history of trauma; it was not his brain. And unlike with the brain antecedent, most participants felt that character remained relevant in determining how people with such experiential histories went on to behave. In other words, whereas the person with a brain anomaly “couldn’t help it,” the person with an abused childhood could have decided, at the last moment, not to be violent.

Monterosso, Royzman, and Schwartz (2005) labeled this pattern of responses “naïve dualism.” Acts are caused either by (mental) intentions or by the working of the physical machinery that is our brains, and these two types of causes are categorically distinct (and see Monterosso and Schwartz 2020 for an extension of this distinction to approaches to understanding addiction). People are responsible for intentional acts but not for acts that their brains “make” them do. It is just this dualism, I suggest, that has made the courts so responsive when it comes to changing penalties for juveniles.

I think the distinction between causal and intentional explanations of action is essential, but I also think that in focusing on brains and neural processes, naïve dualism is paying attention to the wrong things. “Was it biological or psychological?” is the wrong question to be asking in trying to assign responsibility for an action. The right question to be asking is “how strong was the relation between cause and effect, whatever the cause may have been?” In our studies, what people should have been focused on is whether 10, 50, or 80% of people influenced by either cause (brain malfunction or abusive childhood) committed acts of violence. A weak causal relation (10%) sustains the intentional stance whereas a strong one threatens it. Instead, people focused on *what* the cause was and not on how strong it was.

Thus, it seems as though “brain” explanations have an especially strong hold on us. This is an example of idea technology with enormous potential to change how we think about ourselves and others, and to change our view of what our social institutions should do to regulate behavior. Moreover, if dualism is false, as I think it is, this particular piece of idea technology constitutes ideology.

In a related experimental finding, Vohs and Schooler (2008) had participants read text that encouraged a belief in causal determination of human behavior or a text that was neutral regarding the causation of human behavior. The causal determinism text focused on how genes and brain controlled behavior. After reading the text, participants did a series of arithmetic problems in which a “computer glitch” allowed them to cheat. People who read text about causal determinism were more likely to cheat than those who did not. In a second study, people who read text about causal determinism and then took a test that they scored themselves were more likely to inflate their scores than people who did not read the text. Thus, in both studies, suggesting to people that they are not in charge of their actions seemed to remove inhibitions of immoral behavior, as if they were saying to themselves that “the devil (or laws of nature) made me do it.”

There is some reason to doubt the robustness of the Vohs and Schooler (2008) result. Two recent efforts to replicate their findings have been largely unsuccessful (Embley, Johnson, and Giner-Sorolla 2015; Buttrick et al. 2008). However, we can see a pattern of behavior that is conceptually similar with regard to gender differences in mathematical ability. In a clever experiment, Dar Nimrod and Heine (2006) gave young women three sections of a Graduate Record Exam—a math section, a reading comprehension section, and another math section, in that order. In the reading comprehension section, one group of women read a passage suggesting that gender differences in math ability were innate (genetic) and unmodifiable. Another group read a passage about gender differences in math as the product of experience. A third group read a passage about something unrelated to gender and math. How did these three groups do on the second math section? The answer is that the women who read about unmodifiable gender differences in math did substantially worse than the other two groups, which were not distinguishable from each other. Thus, believing the (untrue) ideology that you cannot get smarter in math *can* make that ideology self-fulfilling.

In still another series of studies, Weisberg et al. (2008) had participants judge psychological explanations that did or did not contain neuroscience information that was, in fact, irrelevant to the explanation. Explanations containing neuroscience information were judged as more satisfying. In a second study, participants directly compared good and bad explanations. They were generally successful at selecting the good explanation except when the bad explanation contained neuroscience and the good one did not. A third study showed that neuroscience jargon was unnecessary; people were seduced by explanations that contained any reference to the brain. These results confirm that neuroscience information exerts a seductive effect on people’s judgments, which may explain the appeal of neuroscience information within the public sphere.

In a follow-up, Weisberg, Taylor, and Hopkins (2015) had participants read descriptions of psychological phenomena. Each phenomenon was followed by one of four types of explanation, constructed by crossing explanation quality (good or bad) with neuroscience information (present or absent). Crucially, the neuroscience information was irrelevant to the logic of the explanations and even made the good explanations worse, according to the ratings of experts. When the explanations contained neuroscience information, ratings were significantly higher than when they did not. This was especially true for the bad explanations. That is, non-experts judged that psychological phenomena are explained better with the language of neuroscience.

## Conclusion

In his book, *A Conflict of Visions*, Thomas Sowell (1987) distinguishes between what he calls “constrained” and “unconstrained” visions of human nature. The constrained vision, exemplified by philosopher Thomas Hobbes, focuses on the selfish, aggressive, dark side of human nature, and assumes that we cannot change human nature but must instead impose constraints through an all-powerful state, the Leviathan. The unconstrained vision, perhaps best exemplified by Jean-Jacques Rousseau, sees enormous human possibility and condemns the state for subverting all that is good in human nature.

I think that both Hobbes and Rousseau are wrong. “Nature” dramatically underspecifies the character of human beings. Within broad limits, we are what society expects us to be. If society asks little from us, it gets little. It is clear that, under these circumstances, we must be sure that we have arranged rules and incentives in a way that induces people to act in ways that serve the objectives of the rule makers and the incentive setters. If society asks more of us, and arranges its social institutions appropriately, it will get more. As anthropologist Clifford Geertz (1973) observed, human beings are “unfinished animals.” What we can reasonably expect of people depends on how our social institutions “finish” them.

The idea technology that dominates our age is a fiction; it is ideology. But it is a powerful fiction, and it becomes less and less fictional as it increasingly pervades our institutions. We should not expect this fiction to die of natural causes. To extinguish it, we must nourish the alternatives. And that will not be easy.
